# Different Approaches to Modulation of Microglia Phenotypes After Spinal Cord Injury

**DOI:** 10.3389/fnsys.2019.00037

**Published:** 2019-08-27

**Authors:** Elvira Akhmetzyanova, Konstantin Kletenkov, Yana Mukhamedshina, Albert Rizvanov

**Affiliations:** ^1^OpenLab Gene and Cell Technologies, Institute of Fundamental Medicine and Biology, Kazan Federal University, Kazan, Russia; ^2^Department of Histology, Cytology and Embryology, Kazan State Medical University, Kazan, Russia

**Keywords:** microglia, phenotypes, modulation, spinal cord injury, neuroregeneration

## Abstract

Microglial cells, which are highly plastic, immediately respond to any change in the microenvironment by becoming activated and shifting the phenotype toward neurotoxicity or neuroprotection. The polarization of microglia/macrophages after spinal cord injury (SCI) seems to be a dynamic process and can change depending on the microenvironment, stage, course, and severity of the posttraumatic process. Effective methods to modulate microglia toward a neuroprotective phenotype in order to stimulate neuroregeneration are actively sought for. In this context, available approaches that can selectively impact the polarization of microglia/macrophages regulate synthesis of trophic factors and cytokines/chemokines in them, and their phagocytic function and effects on the course and outcome of SCI are discussed in this review.

## Introduction

Spinal cord injury (SCI) is characterized by numerous pathologic reactions that involve every cell type of the central nervous system (CNS). The activation of microglial cells, which are the first to respond to nervous tissue damage, is one of the essential events of posttraumatic reactions (Gensel and Zhang, 2015). Activated microglia can synthesize not only trophic biomolecules such as neurotrophins, glutamate transporters, and antioxidants, but also effectors such as nitric oxide (NO) and pro-inflammatory cytokines that can be potentially neurotoxic (Persson et al., 2005; Lai and Todd, 2006; Hellwig et al., 2013). In addition to synthesis of many biomolecules, a phagocytic function of microglia is critically important also due to its essential for the removal of degenerating/lost neurons and neuroglial cells, and rearrangement or destruction of synaptic connections (Chen and Trapp, 2016; Jin and Yamashita, 2016; Wolf et al., 2017). Previous studies have shown that the activation of microglia is not a single phenomenon and that there are several different “states of activation” when microglia can have a selective neurotoxic or neuroprotective effect. Given the diversity of microglia functions, the M1/M2 paradigm is a simplified model that reflects two opposite effects on inflammatory responses. However, one should take into consideration that the microglia microenvironment *in vivo* is diverse and its phenotype may rarely shift directly to the other state.

## Microglia Phenotypes

To date, several states of microglia polarization have been described: they are classic activation (M1), alternative activation (M2a), alternative type II activation (M2b), and acquired deactivation (M2c). A number of investigators question whether microglia can acquire an M3 phenotype (Malyshev and Malyshev, 2015; Walker and Lue, 2015). A number of principal studies have identified which markers are specific for classically or alternatively activated microglia (Martinez et al., 2006; Martinez et al., 2013).

M1 microglia are capable of producing active oxygen species that promote a respiratory burst, as well as produce cytokines such as tumor necrosis factor-α (TNF-α), IL-1β, IL-6, and IL-12, thereby mediating inflammatory tissue damage (Liu et al., 2018). M1 microglia are involved in secondary damage after SCI, producing proinflammatory molecules and the formation of a glial scar, which, in turn, creates an environment at the site of injury that is adverse for neuroprotection. Therefore, this phenotype is commonly referred to as neurotoxic (Shechter and Schwartz, 2013; Fan et al., 2016). The phagocytic activity was shown to be inhibited in M1 polarization (Durafourt et al., 2012); at the same time, M1 microglia regulate synaptic pruning and labeling synapses for phagocytosis (Schafer et al., 2012).

Alternative activation is subdivided into two subcategories: M2a and M2b. M2à microglia are considered to respond to IL-4 and IL-13; to have an increased phagocytic activity; to produce an insulin-like growth factor-1, trophic polyamines, and anti-inflammatory cytokines such as IL-10; and to express G-CSF, GM-CSF, and CD209 (Martinez and Gordon, 2014; Franco and Fernandez-Suarez, 2015; Peferoen et al., 2015). The microglia of this type can eliminate cellular debris and stimulate tissue regeneration. M2b microglia are induced by ligation of immunoglobulin Fc-gamma-receptors that results in IL-12 expression, increased IL-10 secretion, and HLA-DR expression. This phenotype is also characterized by active phagocytosis and an increased expression of CD32 and CD64, which are detected in the cerebral microglia in Alzheimer’s disease (Peress et al., 1993). M2c (acquired deactivation) polarization can be caused by the anti-inflammatory cytokine IL-10 or glucocorticoids, an increased expression of transforming growth factor (TGF), sphingosine kinase (SPHK1), and CD163, a membrane-bound receptor for haptoglobin/hemoglobin complexes (Wilcock, 2014). The polarization of microglia/macrophages toward the M2 phenotype occurs to resolve inflammation and degeneration as a whole; thus, this phenotype is characterized as neuroprotective. It is worth noting, however, that although the M2 phenotype of microglia/macrophages plays a positive role in neuroregeneration processes, it has an absolutely opposite role in the case of neoplastic processes in the CNS and has a pro-tumor action (Wu and Watabe, 2017).

A similar pattern of polarization is involved in peripheral macrophages that actively migrate after injury when the blood–brain barrier is damaged. It should be noted that most researchers do not distinguish between microglia and macrophages, subsuming them into the same cell population and using pan markers for their identification. This might be due to the lack of highly specific markers for resident microglia and macrophages migrating toward a site of injury (Franco and Fernandez-Suarez, 2015; Martin et al., 2017).

## Behavior of Microglia/Macrophages in Spinal Cord Injury

It has been previously shown that microglia are activated within the first 24 h after SCI. In the acute period, polarization shifts primarily toward M1 microglia, which release proinflammatory cytokines and chemokines. This results in progression of inflammatory processes after primary mechanical injury (Lee et al., 2009; Nakajima et al., 2012). Shortly thereafter (2–3 days post-injury, dpi), blood monocytes that subsequently differentiate into macrophages phenotypically and morphologically indistinguishable from activated microglia migrate toward the site of injury (Donnelly and Popovich, 2008; Beck et al., 2010). The appearance of M2 microglia/macrophages and their secretion of anti-inflammatory cytokines and chemokines results in inhibiting excessive inflammatory reactions around the site of injury and stimulating regeneration of damaged spinal cord tissues (Gratchev et al., 2008; Varnum and Ikezu, 2012; Shechter and Schwartz, 2013; Weisser et al., 2013). M2 microglia/macrophages are shown to possess an increased phagocytic activity that promotes clearance of posttraumatic debris, leading to accelerated demyelination and resolution of the initial traumatic events (Redondo-Castro et al., 2013; Lampron et al., 2015; Orihuela et al., 2016; Akhmetzyanova et al., 2018).

The primary phase of microglia/macrophage activation peaks on 7 dpi; microglia are reactivated after 14 dpi and then peak on 60 dpi and remain for up to 180 dpi (Beck et al., 2008; Conta and Stelzner, 2008; Kigerl et al., 2009; Bellver-Landete et al., 2019). M1 and M2 microglia/macrophages co-exist at the injury site within the 1st week after SCI, with M1 cells prevailing. However, other researchers have demonstrated that there were no M2 cells and the population of M1 cells significantly decreased after 28 dpi (Kigerl et al., 2009; Francos-Quijorna et al., 2016). These results confirm data on the population of ED1^+^ phagocytic macrophages/microglia, which peak by day 7 after SCI, significantly decreasing by 28 dpi and abruptly increasing again by 90 dpi (Beck et al., 2010). At the same time, Bellver-Landete et al. (2019) showed that activated, proliferating microglia play an important role in the healing process, having a positive effect on tissue sparing and functional recovery after SCI, and this effect persists for 5 weeks after SCI.

These phases of microglia/macrophage activation in SCI can be paralleled with changes observed in the population of macrophages when other tissues and organs are damaged. For example, at the end of the remodeling phase when the main healing processes are completed, macrophages are deactivated, and inflammation resolves. The behavior of microglia/macrophages whose number reduces significantly though variably by 2–4 weeks after SCI is possibly the same. With effective healing, the level of macrophages in non-nervous tissues returns to normal within several weeks after injury in parallel with its healing. On the contrary, wounds that do not heal within 3 months result in a stable activation of macrophages that is a distinctive feature of chronicity (Sindrilaru et al., 2011). In turn, we observed a similar picture during the second phase of microglia/macrophage activation that seems to be triggered by ongoing neurodegeneration in response to which re-activation of these cells prevents a subsequent loss of function.

The polarization of microglia/macrophages after SCI seems to be a dynamic process and can be altered depending on the microenvironment, the stage of the posttraumatic process, and its severity (Kigerl et al., 2009; David and Kroner, 2011). This phenomenon has been demonstrated in several studies and has shown that the behavior of microglia/macrophages depended on the factors of activation, in particular, the type of cells that activated them and the specific activating molecule (Nakajima and Kohsaka, 2002; Nakajima and Kohsaka, 2004; Stout and Suttles, 2004; Shaked et al., 2005; Lai and Todd, 2006; Nakajima et al., 2006; Menzies et al., 2010). An effective method to modulate microglia toward a neuroprotective phenotype in order to stimulate neuroregeneration is actively sought for in addition to investigation into the factors of activation. For this purpose, new approaches are being developed and different biomolecules potentially possessing a selective effect on the polarization of microglia/macrophages regulate their synthesis of trophic factors, cytokines/chemokines, and a phagocytic function tested. The latter can be achieved by affecting the signaling pathways that control microglia activation and polarization, discussed in the following section.

## Microglial Signaling Pathways

**FIGURE 1:**
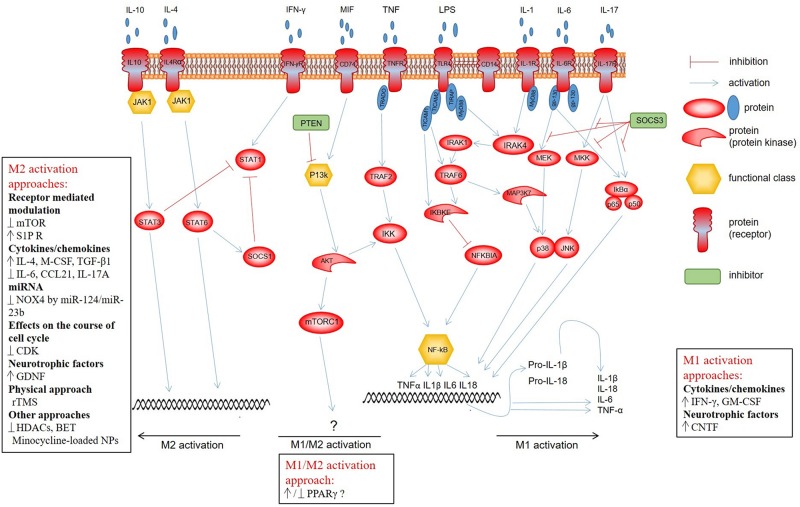
Microglial signaling pathways determining development of cells with divergent abilities. Classical activation of the NF-κB signaling is initiated by TLRs, as well as other cell surface receptors, including specific IL-1 and TNF, and provides M1 polarization of microglia. PI3K/Akt/mTOR is triggered through the CD74 receptor, whose activation is promoted by MIF. There is quite contradictory evidence that this pathway affects the shift of the microglia/macrophage phenotype toward M1 or M2. Anti-inflammatory cytokines IL-10 and IL-4 induce STAT3 and STAT6 phosphorylation, respectively, via JAK1, which promotes polarization toward the M2 phenotype. The activation of STAT1, in turn, leads to polarization toward a neurotoxic M2 phenotype of microglia. Normally, there is a balance between the activation of STAT1 and STAT3/STAT6 that strictly regulates the microglia polarization and activity.

It is now increasingly evident that there are various ways of activation for microglia that determine the generation of cells with divergent abilities (Figure 1). Toll-like receptors (TLRs) are a class of transmembrane receptors involved in the activation of cell-mediated immune response. Out of more than 10 TLRs, identified in both rodents and humans, microglia express at least 9 TLRs along with their adapter proteins (Laflamme et al., 2001; Bsibsi et al., 2002; Dalpke et al., 2002; Olson and Miller, 2004; Zhang et al., 2013). Previous studies have demonstrated TLR-dependent microglia activation in neurodegenerative disorders and different types of CNS injury (Heneka et al., 2005; Fernandez-Lizarbe et al., 2009; Song et al., 2011; Yao et al., 2013). A classical/canonical activation of the nuclear factor κB (NF-κB) signaling, which is essential for both acute and chronic inflammatory responses, is initiated by TLRs, as well as other cell surface receptors, including those for IL-1 and TNF (Shih et al., 2015; Noort et al., 2015). The activated NF-κB allows translocation to the nucleus that results in production of anti-inflammatory cytokines, release of reactive oxygen species (ROS), and microglia modulation toward the M1 phenotype (Pan et al., 2010; Taetzsch et al., 2015; Zhang et al., 2017). The activation of NF-κB transcription factors also plays a key role in neurogenesis, synaptic plasticity, and protection of neurons (O’Riordan et al., 2006; Ahn et al., 2008; Koo et al., 2010). Therefore, NF-κB should be selectively inhibited in microglia and possibly in astrocytes in order to neutralize its neurotoxic role and maintain the neuroprotective one (Brambilla et al., 2005; Crosio et al., 2011; Frakes et al., 2014).

In the presence of inflammation, microglia are activated by means of phosphorylation of p38 mitogen-activated protein kinase (p38/MAPK) and extracellular signal–regulated kinases (ERKs), thereby enhancing phagocytosis, chemotaxis, and the expression of proinflammatory cytokines (Wang et al., 2011; Fan et al., 2017). At the same time, the phosphorylation of p38/MAPK inhibited ULK1 kinase activity and reduced autophagy, allowing the full induction of the inflammatory process during microglia activation (He et al., 2018). The activation of glial cells and the p38/MAPK signaling pathway was demonstrated to be involved in the development of a chronic neuropathic pain that affects up to 80% of patients with SCI (Finnerup et al., 2001; Crown et al., 2008; Detloff et al., 2008). Therefore, p38/MAPK inhibitors are intensively used to reduce activation of the spinal microglia, to prevent/reverse the neuropathic pain symptoms and neuroinflammation in general (Rojewska et al., 2014; Cheng et al., 2015; Kim et al., 2016; Taves et al., 2016).

A phosphatidylinositol 3-kinase (PI3K)/protein kinase B (Akt)/mammalian target of rapamycin (mTOR) signaling pathway that is involved in neuropathic pain progression, as well as astrocyte and microglia activation, is known. Its inhibition reduces the ability of microglial cells to migrate and their number in the site of neurodegeneration (Guo et al., 2017). PI3K/Akt/mTOR is triggered through the CD74 receptor, whose activation is promoted by a macrophage migration inhibitory factor (MIF). The use of MIF suppresses the microglia M1 activation and mitigates the severity of secondary injury around the lesion site in the murine dorsal hemisection model of SCI (Emmetsberger and Tsirka, 2012). At the same time, there is quite contradictory evidence that this pathway affects the shift of the microglia/macrophage phenotype toward M1 or M2 stages (Wang G. et al., 2015; Wang et al., 2016). Therefore, the role of the PI3K/Akt/mTOR pathway in microglia activation and neuroregeneration as a whole following SCI is still controversial (Kanno et al., 2012; Chen et al., 2016). Some researchers relate this to the possibility of isoform-specific cross-talk between PI3K, Akt, and mTORC (Vergadi et al., 2017).

There are natural (phosphatase and tensin homolog deleted on chromosome 10, PTEN) and artificial (ZSTK474, NVP-BEZ235, LY294002, PI828, etc.) inhibitors of the PI3K/Akt/mTOR signaling pathway. PTEN is a lipid and protein phosphatase that has dual substrate specificity and serves as the main negative regulator of PI3K and the PI3K/Akt/mTOR signaling pathway by converting phosphatidylinositol (3,4,5)-trisphosphate (PIP3) into phosphatidylinositol (4,5)-biphosphate (P1P2). In a model of chronic peripheral nerve injury, the PTEN gene overexpression resulted from its delivery with an adenoviral vector (Ad5-PTEN) in the spinal cord. It significantly reduced activation of microglia and astrocytes and prevented a neuropathic pain (Huang et al., 2015). At the same time, such microglia modulation in neurotrauma therapy can negatively affect regeneration as the PTEN expression has been shown to be able to attenuate neuroprotection and lead to an impairment of axonal growth in particular (Zukor et al., 2013; Ohtake et al., 2014; Yin et al., 2018). Thus, the system regulation at the level of this enzyme is a quite dangerous process leading to disarrangement of oppositely directed processes.

A Janus tyrosine kinase (JAK)/signal transducer and activator of transcription (STAT) signaling pathway is one of the most important cascades triggered in response to many modulators of inflammation. Most studies focused on activation of the JAK/STAT3 signaling pathway in the case of neoplastic activity of microglia/macrophages (Zhang et al., 2009; Zhang et al., 2011; Oliva et al., 2012; Koscsó et al., 2013; Popiolek-Barczyk and Mika, 2016). As for microglia/macrophage modulation after SCI, anti-inflammatory cytokines IL-10 and IL-4 induce STAT3 and STAT6 phosphorylation, respectively, via JAK1, and promote polarization toward the M2 phenotype (Koscsó et al., 2013; Wang et al., 2014; Cianciulli et al., 2015; Popiolek-Barczyk and Mika, 2016). Activation of STAT1 and NF-κB transcription factors leads, in turn, to polarization toward a neurotoxic phenotype of microglia/macrophages. There is normally a balance between the activation of STAT1 and STAT3/STAT6 that strictly regulates the microglia/macrophage polarization and activity.

STAT3 is recognized as the main mediator of IL-6 and IL-17 functions (Camporeale and Poli, 2012). There are two main types of IL6 signalization: pro-inflammatory and anti-inflammatory. In microglia, the IL-6 pro-inflammatory signaling pathway is carried out through gp-130, which acts as an antagonist sequestering IL-6 (Hodes et al., 2016). Results obtained by Ma et al. (2010) indicate that SOCS3 can function as a negative regulator of NF-κB, p38 MAPK, and JNK signaling; moreover, an important role for SOCS3 in the regulation of IL-17 and IL-6/R-dependent induction of IL-6 was elucidated. Guerrero et al. (2012) demonstrated that classical microglia activation in mouse SCI could be inhibited by IL-6 blockade. Redondo-Castro et al. (2013) used glibenclamide, an inhibitor of ATP-sensitive potassium channels K_ATP_ to relieve a neuropathic pain in rats with SCI. The inhibition of IL-17, as a mediator of microglia activation, when injecting hyperforin enabled microglia polarization toward the M2 phenotype (Ma et al., 2018). Although studies to inhibit individual mediators of microglia activation demonstrate certain positive changes in some neurodegenerative disorders, they cannot completely neutralize the neurotoxic potential of these cells that is related to their possible activation by means of a common regulator in a signaling cascade.

## Different Approaches to Modulation of Microglia/Macrophages

Supplementary Table 1 contains published data available on the different approaches to modulation of microglia/macrophages *in vitro* and *in vivo*.

### Receptor-Mediated Modulation

Peroxisome proliferator-activated receptor (PPARγ) is a key regulator of the microglia/macrophage M2 phenotype. It is a nuclear receptor capable of modulating inflammatory processes and controlling lipid and lipoprotein metabolism as well as glucose homeostasis (Chinetti et al., 2003; McTigue, 2008). PPARγ was shown to be immediately induced in monocyte differentiation into macrophages (Chinetti et al., 1998). In addition, activation of PPARγ signaling can suppress an inflammatory response by inhibiting NF-κB (Zolezzi et al., 2017). Therefore, PPARγ is described as the main anti-inflammatory regulator of macrophages (Ahmadian et al., 2013). Han et al. (2017) demonstrated that 6-Shogaol, a pungent constituent extracted from *Zingiber officinale* Roscoe can enhance PPARγ expression. Also, the addition of 6-Shogaol to an *in vitro* microglia culture could reduce the lipopolysaccharide-induced (LPS) expression of proinflammatory factors TNF-α, IL1β, IL6, and PGE2 as measured using ELISA. The use of a PPARγ agonist, rosiglitazone, was shown *in vitro* with PCR and flow cytometry to lead to monocyte modulation toward the M2 phenotype (Bouhlel et al., 2007). Additionally, in a murine brain injury model intravenously injected PPARγ activator, malibatol A, an anti-oxidant extracted from *Hopea hainanensis*, could shift the microglia phenotype toward M2 (Pan et al., 2015). Wen et al. (2018) compared the effects of intraperitoneal injections of the PPARγ agonist, rosiglitazone, and the PPARγ antagonist, GW9662, in a lateral fluid percussion injury model in mice. Using ELISA, real-time PCR and immunohistochemistry, they observed that 72 h after injury, expression levels of proinflammatory cytokines TNF-α, IL-1β, and IL-6 were significantly higher and that of anti-inflammatory IL-10 was lower in the group treated with GW9662. Further *in vitro* experiments in a primary culture of mouse microglia were conducted and results demonstrated that rosiglitazone increased the expression of M2 markers (CD206 and YM-1) and decreased that of M1 markers (TNF-α, IL-6, IL-1β, and IL-10). Interesting results were obtained by Park et al. (2007) using pioglitazone in a rat model of SCI. They showed that intraperitoneal injection of pioglitazone causes a decrease in the number of reactive macrophages, attenuates myelin loss, and improves functional recovery from SCI. The results of this study were confirmed by McTigue et al. (2007), where the rat electromagnetic SCI model again showed pioglitazone’s ability to reduce the number of activated phagocytic microglia by 7 dpi.

Neuropeptides Y (NPY), Y1 receptor activators, suppress the innate immune response by reducing the release of interleukin-1β and NO, migration, and phagocytosis of activated microglial cells (Farzi et al., 2015). NPY were demonstrated to significantly restrain the microglia activation by inhibiting LPS-induced Fc-receptor-mediated phagocytosis (Ferreira et al., 2012). Macrophage antigen complex-1 receptor (MAC1R), a molecule mediating the macrophage activation in response to various stimuli, plays an important role in phagocytosis (Le Cabec et al., 2002). In fact, MAC1R is a key receptor for both toxins and classic acute-phase reactants such as fibrinogen, which activate microglia/macrophages manifesting in enhanced phagocytic activity and a release of ROS (Adams et al., 2007; Pei et al., 2007). The investigation of the role that NPY and MAC1R play in regulation of phagocytic activity of microglial cells is promising and requires further research.

Rapamycin, an inhibitor of the mTOR receptor in mammals, is involved in numerous cellular processes such as neuroprotection in neurodegenerative disorders (Ravikumar et al., 2004; Malagelada et al., 2010) and neuroregeneration after cerebral injury and SCI (Erlich et al., 2007; Kanno et al., 2012). Rapamycin inhibits the mTOR pathway by preventing the activation of p70S6K protein kinase (Schmelzle and Hall, 2000). Rapamycin neuroprotective properties are due to its ability to stimulate autophagy (Ravikumar et al., 2004; Malagelada et al., 2010). It is also involved in suppression of microglia activation and reduction of inflammation in the CNS by selective inhibition of the mTORC1 complex (Russo et al., 2009). *In vivo* studies using rat SCI model showed the ability of rapamycin to attenuate microglial activation and neuroinflammation processes by reducing the number of M1 cells and, as a result, TNF-α production (Chen et al., 2013; Song et al., 2015). However, Eldahan et al. (2018) caution against the use of rapamycin as a therapeutic intervention for SCI due to its toxic effects and exacerbation of cardiovascular dysfunction.

FTY720 is an agonist of the S1P receptor and a derivative of ISP-1 (myriocin), a metabolite of the Chinese fungus *Iscaria sinclairii*, as well as a sphingosine structural analog. This is a novel immunomodulator that promotes transplantability in numerous models by inhibiting lymphocytes. FTY720 plays the role of a switch in the polarization of microglia from M1 to M2 through the STAT3 protein activation that has been established in a white matter ischemic injury model (Qin et al., 2017). The results obtained also provide evidence that FTY720 has a protective effect against structural damage to the nodes of Ranvier and demyelination after hypoperfusion. It should be noted that, in some cases, FTY720 was effective in treating SCI but did not affect the activation of microglia/macrophages (Norimatsu et al., 2012; Wang J. et al., 2015).

### Cytokines/Chemokines

Cytokines play an important role in posttraumatic processes; in particular, microglia cells can influence healing by controlling levels of some of them. Activating and blocking agents to modulate inflammatory processes for most of these cytokines are under development. For example, IL-4 is considered the strongest polarizing cytokine for M2a microglia response. In the IL-4R-deficient mice SCI model, there was a decrease in the production of anti-inflammatory cytokines such as arginase, IL-1, and CCL2, which indicates the predominance of M1 microglia (Fenn et al., 2014). A single intraspinal injection of IL-4 48 h after SCI was shown to be sufficient to switch the microglia phenotype toward M2 and, what is more important, it was associated with improved functional recovery in mice with an SCI (Francos-Quijorna et al., 2016). Interestingly, in response to exposure, IL-4 can modulate the morphology of microglia *in vitro* from amoeboid (activated) to a more ramified (quiescent) one associated with a more activated phenotype; at the same time, IFN-γ and GM-CSF have the opposite effect (Rostam et al., 2017).

IL-6 is a key factor triggering inflammation after SCI; it also promotes microglia M1 activation (Bethea and Dietrich, 2002). An IL-6 blocking agent—monoclonal anti-mouse receptor antibody IL-6 (MR16-1)—administered into the site of SCI in mice promoted alternative M2 microglia activation and resulted in improved tissue integrity as well as an increased number of myelinated fibers (Guerrero et al., 2012). Moreover, inhibition of the EGFR/MAPK pathway that suppresses microglia activation and associated cytokine production decreases neuroinflammation-related secondary damage and thereby provides neuroprotection in rats after SCI (Qu et al., 2012). It is thought that EGFR can be a therapeutic target and inhibitors C225 and AG1478 have the potential to be used in the treatment of SCI (Qu et al., 2012). Chemokine CCL21 neutralization was also shown to reduce microglia M1 activation and to cause neuronal hyperexcitability of lateral posterior thalamic nuclei (Zhao et al., 2007).

IL-6 is a key cytokine accelerating the IL-17 production (Camporeale and Poli, 2012). IL-17 is a well-known proinflammatory cytokine associated with M1 activation of microglia (Kim and Moalem-Taylor, 2011; Zong et al., 2014). The inhibition of IL-17 as a mediator of microglia activation after hyperforin injection promoted microglia polarization toward the M2 phenotype in a murine acute cerebral mechanic trauma model (Ma et al., 2018). TGF-β1 is a polypeptide component of a transforming cytokine factor. It was shown in an *in vivo* murine stroke model that cerebro-ventricular injections of TGF-β1 promoted microglia M2 activation as well as improvement of functional recovery in mice (Taylor et al., 2017).

### MicroRNA

Lately, special emphasis is being given to the role that miRNA plays in the pathogenesis of many diseases including diseases of the CNS. It has been shown that miRNA administration can be an effective therapeutic approach to the management of neurodegenerative processes. MiRNAs regulate the expression of a great number of genes by stimulating RNA interference pathway degradation or by preventing the translation of target genes. High miR-124 levels were reported in resident cerebral and spinal microglia, as well as their activation *in vitro* and *in vivo* to promote a decreased miR-124 expression (Ponomarev et al., 2011). miR-124 is considered to regulate the activity of microglia/macrophages by down-regulating the expression of CCAAT-enhancer-binding protein-α, a transcription factor regulating the differentiation of myeloid cells. Therefore, high miR-124 levels are thought to be required to maintain microglia in a quiescent state. Willemen et al. (2012) demonstrated that intrathecally injected miR-124 promoted the maintenance of microglia in this quiescent state and alleviated chronic posttraumatic processes in the spinal cord of rats with hyperalgesia. Im et al. (2012) reported similar results when injecting miR-23b intrathecally to mice in a neuropathic pain model. Based on their results, a return of miR-23b to normal levels decreased the expression of inflammatory proteins, reduced the number of Iba1^+^ cells in the spinal cord tissue, and alleviated a neuropathic pain resulting from SCI.

### Cell Cycle Modulation

It was found that exposure on a cell cycle course can also modulate cell phenotype. There were changes in cell cycle course following SCI and effects of systemic administration of delayed (24 h) flavopiridol, an inhibitor of major cyclin-dependent kinases, on functional recovery and histopathology in a rat SCI model (Wu et al., 2012). The treatment with flavopiridol attenuated the number of Iba-1^+^-microglia in the intact tissue and, as a result, increased the myelinated area of the white matter. Moreover, flavopiridol attenuated the expression of Iba-1 and glactin-3, associated with microglia M1 activation and astrocyte reactivity by reducing the GFAP, NG2, and CHL1 expression.

### Neurotrophic Factors

Although there are numerous approaches to modulate microglia in the CNS, the search for new approaches to effective polarization strictly toward a neuroprotective phenotype and introduction of results into clinical practice is still relevant. Neurotrophic factors are molecules that increase the potential of nervous system cells to proliferate, survive, migrate, and differentiate. For instance, a ciliary neurotrophic factor (CNTF) exerts a positive effect on reactive M1 microglia, promoting their survival and activation after intracerebral injection to mice *in vivo* (Kahn et al., 1995). However, Rocha et al. (2012) demonstrated that, on the contrary, a glial cell line-derived neurotrophic factor (GDNF) inhibited the activation of reactive M1 microglia *in vitro* (Rocha et al., 2012).

Selective modulation of microglia with recombinant adenoviruses carrying the GDNF gene *in vivo* seems promising. Zhuravleva et al. (2016) demonstrated that microglia transduction with an adenovirus encoding for GDNF (Ad5-GDNF) promoted a reduced phagocytic activity of these cells. We have conducted a study to evaluate effects of Ad5-GDNF transduction on the morphology and phenotype of microglial cells as well as effects of transplanting these cells on posttraumatic processes in the rat spinal cord. It was shown that microglia transduction with Ad5-GDNF down-regulated expression of CD45, but their transplantation into the site of a rat SCI did not increase the area of intact tissue as compared to similar transplantation of Ad5-EGFP microglia with fair phagocytic activity (Akhmetzyanova et al., 2018).

### Physical Methods

Repetitive transcranial magnetic stimulation of the motor cortex was shown to reduce microglia M1 activation after SCI and alleviated symptoms of neuropathic pain and allodynia (Kim et al., 2013). A method of physical exposure to alleviate SCI consequences is promising as it is non-invasive and has few side effects. However, this approach to treatment is just gaining ground and is not yet fully understood. Therefore a comprehensive assessment of modulation mechanisms triggered by a physical exposure is required.

### Other Approaches

Lipopolysaccharide is an essential molecular component of the outer membrane of gram-negative bacteria and is recognized by an immune system as an invasion marker of bacterial pathogens. LPS is more often used to induce a potent immunological response and activate microglia/macrophages. It was found that pre-conditioning of microglia with LPS 48 h prior to transplantation enabled M2 polarization in mouse SCI (Hayakawa et al., 2014). The results obtained were evaluated by measuring the expression of mRNA markers of M1 (iNOS, CD86, and CD16) and M2 (arginase1 and CD206) microglia.

Histone deacetylases (HDACs) are proteins targeted to remove acetyl groups from lysine residues of target proteins. HDAC3 is most commonly found in the brain and is a regulator of inflammatory processes (Broide et al., 2007). HDAC3-deficient macrophages have a reduced ability to activate the expression of inflammatory genes in response to LPS stimulation (Chen et al., 2012). At the same time, it was found that HDAC3 is an epigenomic brake in macrophage alternative (M2) activation (Mullican et al., 2011). Malvaez et al. (2013) used protein mass spectrometry in the study *in vitro* to detect global molecular changes in resident microglia exposed to RGFP966, a selective HDAC3 inhibitor, by investigating a signaling pathway through which RGFP966 regulated an inflammation. They observed that RGFP966 could inhibit TLR and STAT3/5 signaling pathways of microglia M1 activation and that this resulted in an anti-inflammatory microglia response manifesting as a reduced expression of proinflammatory cytokines such as IL-6 and TNF-α (Xia et al., 2017). This is confirmed by another *in vitro* study, where it was shown in a primary culture that treatment with HDAC inhibitors promoted suppression of the innate immune activation of microglia (Kannan et al., 2013). A similar study demonstrated that HDAC3 arrest with the same selective inhibitor RGFP966 facilitated the shift toward an anti-inflammatory microglia response that resulted in gaining a neuroprotective phenotype by these cells and improved functional recovery in an SCI model *in vivo* (Kuboyama et al., 2017). Bromodomain and extraterminal (BET) proteins are readers of histone acetylation labels, thereby affecting the transcription of genes and thus playing an important role in regulation the expression of pro-inflammatory cytokine expression (Belkina et al., 2013). Sánchez-Ventura et al. (2019) investigated the influence of BET inhibitor JQ1 in polarizing microglia on bone-marrow-derived macrophage *in vitro* and *in vivo* in SCI mice and showed that JQ1 promotes polarization of microglia toward the M2 phenotype, reducing the expression of pro-inflammatory cytokines IL-6, IL-1β, and TNF-α and increasing the expression of anti-inflammatory cytokines Arg1 and CD206.

It was shown that an early administration of minocycline, a known anti-inflammatory agent, inhibiting poly (ADP-ribose) polymerase-1 (PARP-1), which both promotes cell death and inhibits microglia activation and an inflammation in general, can reduce a degree of neuronal hyperexcitability for up to 4 weeks after SCI (Alano et al., 2006; Tan, 2009). In addition, minocycline-loaded polymeric nanoparticles (NPs) injected into the site of an SCI can selectively target activated microglial cells and modulate their phenotypes toward the anti-inflammatory one by inhibiting PARP-1 and matrix metalloproteinases 2 and 9, which improves the course of secondary traumatic processes in a murine SCI model. The treatment with minocycline-loaded NPs resulted in a reduced activation and decreased proliferation of microglia around the site of injury. As a result, the decreased number of cells with a phagocytic phenotype switched toward quiescent microglia with a low CD68 staining level. The treatment with these particles appeared effective for 15 post-injury days and was related to a prolonged anti-inflammatory stimulus associated with microglia activation (Papa et al., 2013). Another study demonstrated that the administration of minocycline-loaded NPs in an acute period following trauma in a murine SCI model could effectively modulate resident microglial cells from M1 to M2 phenotype, which reduced a proinflammatory response, restored the nervous tissue integrity, and improved behavior test scores for up to 63 post-injury days (Papa et al., 2016).

## Conclusion

Although there are many studies aimed at elucidating mechanisms of microglia/macrophage modulation, their phenotype, and role in various pathologies, currently, no effective methods to modulate microglia toward a neuroprotective phenotype in order to stimulate neuroregeneration are employed in clinical practice. In addition, there is an urgent need to develop a highly specific panel of markers for resident microglia and macrophages migrating to a site of pathology, as well as complete elucidation of every external (specifically activating molecules secreted by surrounding cells) and internal factor (signaling pathways) affecting the modulation of their phenotype.

## Author Contributions

EA was in charge of the collection of data on the approaches to modulation of microglia/macrophages and compilation of a table. KK was in charge of the collection of data on the microglial signaling pathways. YM was in charge of the data collection about the microglia phenotypes and behavior of microglia/macrophages in the area of SCI and Figure 1 drawing. AR was in charge of the article content compilation and manuscript writing.

## Conflict of Interest Statement

The authors declare that the research was conducted in the absence of any commercial or financial relationships that could be construed as a potential conflict of interest.
