# Supplementary material for: Different Approaches to Modulation of Microglia Phenotypes After Spinal Cord Injury
Source: Front Syst Neurosci. 2019 Aug 27;13:37. doi: 10.3389/fnsys.2019.00037 (PMC6718713; doi:10.3389/fnsys.2019.00037)
Supplement: Supplementary file 1 [file Table_1.docx]

| **Cell culture & protocol details** | **Source of cells (in vitro study)** | **Modulator, type of injection** | **Polarization/activation** | **Other reported effects** | **Reference** |
| --- | --- | --- | --- | --- | --- |
| **Receptor mediated modulation** | | | | | |
| Monocyte culture | human atherosclerotic plaques | PPARγ activator (rosiglitazone, 100 nM) | М2 polarization | ↑CD163, CD206, AMAC1, IL-10 | Bouhlel et al., 2007 |
| Microglia culture  Mice, middle cerebral artery occlusion | mice cerebral cortex | PPARγ activator (1μM malibatol A for 15 h. in vitro; malibatol A, 20 mg/kg in vivo, vc. injection) | М2 polarization | in vitro ↓IL-6, iNOS, MCP-1, TNFα;  in vivo ↑CD206;  ↓CD 16/32, TNFα, IL1β, iNOS, IL6 | Pan et al., 2015 |
| Microglia culture  Mice, lateral fluid percussion injury | mice cerebral cortex | PPARγagonist (rosiglitazone, 10 µM) or PPARγ inhibitor (GW9662, 10 µM) for 15 h  PPARγ agonist (rosiglitazone) or PPARγ inhibitor (GW9662), 6 mg/kg, ip. injection) | M1 polarization (GW9662) | in vitro GW9662: ↑TNF-α, IL-6, IL-1β, IL-10;  Rosiglitazone: ↓TNF-α, IL-6, IL-1β, IL-10;  in vivo GW9662: ↑TNF-α, IL-1β, IL-6;  Rosiglitazone: ↓TNF-α, IL-1β, IL6, axonal degeneration | Wen et al., 2018 |
| Rats, contusion SCI, Th9 |  | PPARγagonist (pioglitazone, 0.5, 1.5 and 3 mg/kg, ip. injection) | attenuation of microglial activation | ↓Iba1^+^ and ED1^+^ cells;  ↑myelin preservation | Park et al., 2006 |
| Rats, OSU  electromagnetic SCI, Th8 |  | PPARγagonist (pioglitazone, (10 mg/kg, ip. injection) | attenuation of microglial activation | ↓ED1^+^ cells | McTigue et al., 2007 |
| Microglia culture | mixed cultures of rat cortical glial cells | mTOR inhibitor (RAD001,  rapamycin, range of  0.1–5 nM) | attenuation of microglial activation and  proliferation | ↓NO production, cytokine-dependent microglial activation (RAD001 & rapamycin);  ↓NOS2, COX1 (RAD001 only) | Dello Russo et.al., 2009 |
| Rats, contusion SCI, Th11 |  | mTOR inhibitor (rapamycin, 0.5-1 mg/kg, ip. injection) | attenuation of microglial activation and  neuroinflammation | ↓ED1^+^ cells;  ↓IL1β, TNFα  ↑behavioral recovery | Song et al., 2014  Chen et al., 2013 |
| Microgliaculture | murine N9 microglia cell line | NPY (Y_1_ receptors activator,  1 μM) | induces resting-like morphology in microglia | ↓IL-1b, NO;  prevents microglial cell motility | Ferreiraetal., 2012 |
| Microgliaculture | midbrain tissues of MAC1^-/-^ mouse embryos in 14 postnatal day | deficient in the MAC1 receptor | attenuation of microglial activation | ↓LPS-induced neurotoxicity & ROS; ↓TNFa;  ↓F4/80 (expressed by murine phagocytes) | Pei et.al., 2007 |
| Microglia culture  Mice, chronic cerebral hypoperfusion | brain of neonatal mice | agonist of S1P receptor (FTY720, 100 nmol/L in vitro; 0.3 mg/kg in vivo, ip. injection) | М2 polarization | in vitro ↓IL-1β, TNF-α;  ↑IL-13, TGF-β;  in vivo ↓CD16/32^+^, CD86^+^; ↑CD206^+^, CD23^+^;  ↓damage of Ranvier nodes | Qinet.al., 2017 |
| **Cytokines/chemokines** | | | | | |
| Macrophage culture | bone marrow of CCR4^–/–^ mice | IL-4,  10 ng/mL | М2 polarization | ↑Ym1, H3K4me3 methylation, *Arg1,* FIZZ1; ↓iNOS;  ↓H3K27me2/3 methylation | Ishii etal., 2009 |
| IL-4R-deficient mice, contusion SCI, Th9 |  |  | М1 polarization | ↓arginase, IL-1, CCL2;  ↓behavioral recovery | Fenn et al., 2014 |
| Mice, contusion SCI, Th11 |  | IL-4, 100 ng, is. injection | М2 polarization | ↑CD16/32, CD206;  ↑preservation of neurons, myelin sparing, behavioral recovery | Francos-Quijornaet al., 2016 |
| Macrophage culture | buffy coats from healthy donors | IFN-γ, 20 ng/ml,  GM-CSF, 50 ng/ml;  IL-4, 20 ng/ml,  M-CSF, 50 ng/ml | M1 polarization;  M2 polarization | ↑IL-6, IL-1β, TNFα, *STAT1, SOCS3, IRF5*;  ↑IL-10, IL-1RA, CCL18, *STAT6* | Rostam et al., 2017 |
| Mice, contusion SCI,Тh10 |  | temporal blockator IL-6 (MR16-1,  50 μg/g,  ip. injection) | М2 polarization | ↓iNOS; ↑Arg1;  ↑behavioral recovery, myelin sparing | Guerrero et al., 2012 |
| Rats, contusion SCI, Th9 |  | antibody against CCL21, 5 ng, iVPL injection | attenuation of microglial activation | ↓Cd11b/c;  ↓neuronal hyperexcitability, pain behaviors | Zhao et al., 2007 |
| IL-17a^-^/^-^ mice, focal cerebral ischemia |  | IL-17A inhibitor (hyperforin, 0.5 µg, iv. injection) | М2 polarization | ↓CD16, CD11b, CD32, iNOS, TNFα; ↑IL-10, Arg1, TGFβ, CD206, YM1;  ↓infarct volumes; ↑ behavioral recovery | Ma et al., 2018 |
| BM chimera mice, intracerebral hemorrhage |  | TGF-β1,  10 ng, iv. injection | М2 polarization | ↓IL-6, TNF, gene*Il6;* ↑IL-13;  ↓neuroinflammation, behavioral recovery | Taylor et al., 2017 |
| **miRNA** | | | | | |
| LysM-GRK2^+/−^ mice, IL-1β (200 ng/ml) induced hyperalgesia |  | NOX4 inhibitor  (miR-124, 5 μl,  it. injection) | М2 polarization | ↑CD206, Arg1;  ↓CD16/32;  ↓expression of proinflammatory genes; reversed persistent hyperalgesia | Willemen et al., 2012 |
| Mice, contused SCI induced neuropathic pain*,* Th9-10 |  | NOX4 inhibitor  (miR-23b, 10 mM, is. injection) | attenuation of microglial activation | ↑ED1, COX2, TNFα, IL-1b;  ↓Iba1, ROS;  ↓neuropathic pain | Im et al., 2012 |
| **Effects on the course of cell cycle** | | | | | |
| Rats, contusion SCI, Тh8 |  | CDK inhibitor (flavopiridol,  1 mg/kg, ip. injection) | attenuation of microglial activation | ↓Iba1;↓apoptosis of neurons & oligodendrocytes, inflammation, reactive astrogliosis; ↑myelination | Wu et al., 2012 |
| **Neurotrophic factors** | | | | | |
| Rats, postnatal  day 5 |  | CNTF, 0.25 µg,  g.m. injection just above the lateral ventricle | microglia activation | ↑ED1; ↑astrogliosis | Kahn et. al., 1995 |
| Microglia culture | ventral midbrain of rats in 4 postnatal day | GDNF,  200 ng/ml | attenuation of microglial activation | ↓ROS, ↓phagocytic activity | Rochaet. al., 2012 |
| Microglia culture | brain of neonatal mice | Ad5-GDNF, MOI 40 | attenuation of microglial activation | ↓phagocytic activity | Zhuravleva, 2016 |
| Microglia culture  Rat, contusion SCI, Th8 | brain of neonatal rats | Ad5-GDNF, MOI 40 in vitro  MG+Ad5-GDNF (5 μl suspensions per animal, containing 1×10^6^ cells, is. injection) | attenuation of microglial activation | In vitro ↓CD45;  In vivo ↓nervous tissue integrity | Akhmetzyanova et al., 2018 |
| **Physical approach** | | | | | |
| Rats, contusion SCI, Тh9 |  | rTMS of brain, 25 Hz | attenuation of microglial activation | ↓GFAP, Iba1;  ↓neuropathic pain | Kimet.al., 2013 |
| **Other approaches** | | | | | |
| Microglia culture | brain of neonatal mice | HDACs inhibitor,  (TSA, 30 nM &SAHA,l µM) | М2 polarization | ↓*TNFa, IL-6, iNOS*gene, CD86, CD40 | Kannan, et.al., 2013 |
| Microglia culture  Mice, contusion SCI, Th8 | cerebral cortices of mice in 2 postnatal day | HDAC_3_ inhibitor (RGFP966, 10 μM in vitro;  10 mg/kg in vivo, ip. injection) | М2 polarization | in vitro ↓iNOS; ↑CD206;  in vivo ↑behavioral recovery, axonal preservation | Kuboyama, et.al., 2017 |
| Macrophage culture  Mice, contusion SCI, Th11 | bone marrow | BET inhibitor (JQ1, 1 μM in vitro;  30 mg/kg in vivo, ip. injection) | М2 polarization  attenuation of microglial activation | in vitro ↓iNOS; ↑ARG1, CD206;  in vivo ↓IL-6, IL-1β, TNF-α, Iba1; ↑spared tissue area, behavioral recovery | Sánchez-Ventura et al., 2019 |
| Microglia culture  Mice, induced by injected hydrogel SCI, Тh12 | spinal cord of  mice in 13 postnatal day | minocycline-loaded NPs (0.07 μg/mL in vitro; 0.5 μL/site in vivo, is. injection) | М2 polarization | in vitro ↓IL-1β, TNFαR;  in vivo ↓IL-6, CD68 | Papa et.al., 2013 |
| Microglia culture  Mice, contusion SCI, Тh12 | spinal cord of mice in 13 postnatal day | minocycline-loaded NPs (0.25 mg in vitro, 0.25 mg in vivo,  is. injection) | М2 polarization | in vitro ↓IL-1β, TNFα; ↑Arg1, YM1;  in vivo ↑spared tissue area, behavioral recovery | Papa et.al., 2016 |

ig.-intragastric, is.-intraspinal; it.-intrathecal; ip.-intraperitoneal; iv.-intraventricular; iVPL-intra-ventral posterolateral; vc.- vena caudalis; gm.-gray matter injection; ↑–increase; ↓–decrease;

**Supplementary Table 1. Different approaches to modulation of microglia/macrophages in vitro and in vivo.**
